# Phylogenetic analysis consistent with a clinical history of sexual transmission of HIV-1 from a single donor reveals transmission of highly distinct variants

**DOI:** 10.1186/1742-4690-8-54

**Published:** 2011-07-07

**Authors:** Suzanne English, Aris Katzourakis, David Bonsall, Peter Flanagan, Anna Duda, Sarah Fidler, Jonathan Weber, Myra McClure, SPARTAC Trial Investigators, Rodney Phillips, John Frater

**Affiliations:** 1 Nuffield Department of Clinical Medicine, Peter Medawar Building for Pathogen Research, Oxford University, South Parks Road, Oxford, OX1 3SY, UK; 2 Department of Zoology, Oxford University, South Parks Road, Oxford, OX1 3PS, UK; 3 Division of Medicine, Wright Fleming Institute, Imperial College, St. Mary's Hospital, Norfolk Place, Paddington, London W2 1PG, UK; 4 The James Martin 21st Century School, Peter Medawar Building for Pathogen Research, South Parks Road, Oxford, OX1 3SY, UK; 5 Oxford NIHR Biomedical Research Centre, Oxford, UK

## Abstract

**Background:**

To combat the pandemic of human immunodeficiency virus 1 (HIV-1), a successful vaccine will need to cope with the variability of transmissible viruses. Human hosts infected with HIV-1 potentially harbour many viral variants but very little is known about viruses that are likely to be transmitted, or even if there are viral characteristics that predict enhanced transmission *in vivo*. We show for the first time that genetic divergence consistent with a single transmission event *in vivo *can represent several years of pre-transmission evolution.

**Results:**

We describe a highly unusual case consistent with a single donor transmitting highly related but distinct HIV-1 variants to two individuals on the same evening. We confirm that the clustering of viral genetic sequences, present within each recipient, is consistent with the history of a single donor across the viral *env, gag *and *pol *genes by maximum likelihood and Bayesian Markov Chain Monte Carlo based phylogenetic analyses. Based on an uncorrelated, lognormal relaxed clock of *env *gene evolution calibrated with other datasets, the time since the most recent common ancestor is estimated as 2.86 years prior to transmission (95% confidence interval 1.28 to 4.54 years).

**Conclusion:**

Our results show that an effective design for a preventative vaccine will need to anticipate extensive HIV-1 diversity within an individual donor as well as diversity at the population level.

## Background

A successful HIV-1 vaccine would be designed based upon the antigenicity of transmissible viruses. At the global level, multiple subtypes with evidence of on-going evolution [1] result in a level of diversity that has already frustrated all efforts to synthesize a universal HIV-1 vaccine [2]. Additionally, substantial virus diversity develops within a single host during chronic infection [3], and it is unclear which viral variants are transmissible to a new host. Recent efforts have concentrated on inferring variant transmissibility by characterizing the precise genetic and antigenic features of viruses found during very early stages of infection [4-9].

Single viral variants are detected in a significant proportion of new HIV-1 infections *in vivo*, indicating a profound genetic bottleneck [6,10]. The degree of genetic bottleneck has been associated with the route of transmission [11-13]. Another factor associated with the number of infecting variants is the presence of genitourinary infections [10]. Together, these data suggest that differences in the degree of genetic bottleneck are related to variations in mucosal defence and its integrity.

However, the actual mechanism of this genetic bottleneck remains unclear, and studies may be confounded by variations in both the risk of transmission among donors and the diversity of transmissible virions within donors [9]. The highest risk of transmission occurs during primary infection when the population size of infectious virus peaks [14]. However, viral diversity within the acutely-infected donor is limited, potentially making transmitted viruses indistinguishable in the recipient [4-6,11,15].

Furthermore, genetic analysis has also indicated that mucosal defence and integrity are not the only explanations for the apparent genetic bottleneck. Demographic models have been developed that avoid unsupported prior assumptions about the degree of genetic bottleneck [16]. Viral variability was compared [9] in *gag *and *env *genes after transmission in mother-to-child transmission cases and in men who have sex with men (MSM). Viral variability after transmission was not consistently associated with the route of transmission [9]. In addition, a severe genetic bottleneck may be a sufficient, but not a necessary, condition for random transmission of genetic variability [9].

If transmission of viral variability is not random, then transmission may occur by natural selection [17,18]. However, transmissibility has not yet been associated with specific viral characteristics. Most new, sexually-transmitted HIV-1 infections are CCR5-tropic [4,19], but this may reflect biased representation of these variants in genital fluids [20,21]. In eight cases of heterosexual transmission of subtype C [22], transmitted variants tended to have fewer potential N-linked glycosylation sites (PNLGSs) and shorter hypervariable loops than the average variant in the donor host. In addition, recipient env-pseudotyped virus was more susceptible to neutralization by donor serum than donor env-pseudotyped virus [22]. A study of 35 subtype A cases from Kenya, and 13 subtype B cases from the USA [23] found that recently-infected persons had viruses with shorter, less-glycosylated V1V2 loops compared with a database of viruses [23]. However, studies of subtype B have not shown a consistent decrease in hypervariable loop length or the number of PNLGSs [24,25]. Therefore, there is no firm evidence that natural selection determines transmission of viral variants.

Animal models of HIV infection that use the closely-related simian immunodeficiency virus (SIV) have also demonstrated that many different variants circulating within the host are transmissible. A low-dose, intrarectal inoculum of SIV was given to 18 rhesus macaques [26] to mimic physiological concentrations. Although between one and five variants initiated new infections, the viruses transmitted to all macaques collectively reflected the diversity within the inoculum [26]. Another study [27] demonstrated a stochastic pattern of *V1V2 *variant transmission from an inoculum. Therefore, a broad range of viruses circulating in a single donor may be potentially transmissible at any one time, consistent with the hypothesis that transmission of viral variants is a random process.

To demonstrate that this lack of predictability is also true for HIV-1 transmission in humans, we present an unusual case consistent with a clinical history of one male having transmitted significantly divergent HIV-1 variants to two recipients on the same evening. We show that, as with macaques, diversity in early infection is limited and compatible with transmission of a single variant to each recipient, but also that a single donor can transmit two very different HIV-1 strains contemporaneously. Furthermore, we do not find any evidence that this between-host genetic divergence is evidence of selection pressure from either humoral or cellular immunity during or since transmission. Finally, if transmission is a random process, we hypothesize that a protective vaccine would need to cover the breadth of transmissible variation within individual donors as well as population-wide diversity.

## Results and Discussion

### Case history of a single, third party exposure and recent seroconversion

**Figure 1:**
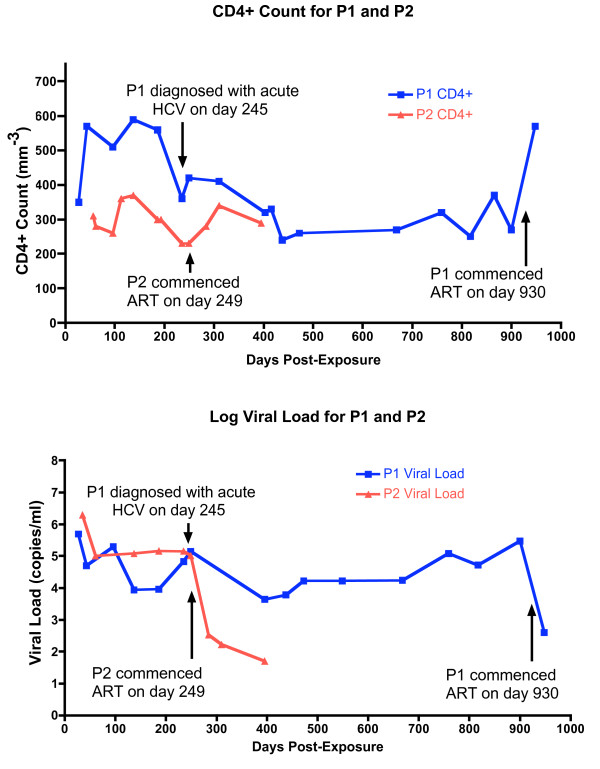
**Clinical data for P1 compared with that of P2**. The **a**. CD4+ counts (/mm^3^) and **b**. log viral loads (copies/ml) for P1 (blue) and P2 (red) are shown. P1 and P2 were exposed to the same third party on day 0. P1 remained off therapy for 930 days post-exposure whilst P2 progressed more rapidly and commenced HAART 249 days post-exposure. Plasma for baseline sequencing was collected on day 63 but the CD4+ count or VL were not recorded. At day 245, P1 was diagnosed with acute HCV infection and had evidence of super-infection in plasma collected at day 235, having been exposed to a fourth person after day 63.

Two adult males, P1 and P2, reported a single sexual encounter each with the same third-party that occurred on day 0 (Figure 1). P1 and P2 reported subsequent exposure only to each other prior to enrolment in the Short Pulse AntiRetroviral Therapy at HIV seroConversion (SPARTAC) trial. Despite repeated efforts, the third-party donor could not be traced. On day 6 post-exposure, P1 presented to his primary care physician with symptoms compatible with HIV seroconversion. On day 25, P1 tested positive for HIV-1 by ELISA with an incident result on a detuned ELISA, suggestive of recent infection [28,29]. P2 had a positive HIV-1 PCR and negative HIV-1 ELISA on day 22, and on day 35 was p24 positive, but negative by Murex ELISA (R&D Systems, UK) [30]. The Murex ELISA was repeated on day 56 and had become clearly positive. Although, the Murex ELISA was positive in P1 earlier than in P2, the result was consistent with reported between-host variability in both the duration of the pre-viraemic phase and the timing of the appearance of markers of seroconversion [30,31]. Therefore, clinical and laboratory evidence supported recent seroconversion in P1 and P2.

P1 and P2 were sampled on the same day when they enrolled in the SPARTAC trial, 63 days post-exposure. Both participants were randomized to receive no therapy. Plasma for sequencing was re-sampled on the same date from both participants, on day 235 post-exposure. P1 reported exposure to a fourth party after day 63 and before day 235. Evidence of HIV-1 super-infection in P1 was seen on plasma collected at day 235 (data not shown). On day 245, P1 was diagnosed with acute hepatitis C virus (HCV) infection (Figure 1) having been negative for HCV by PCR and antibody on day 29. He commenced treatment with ribavirin and interferon after day 245. Therefore, all time-points after day 63 were excluded from further phylogenetic analysis.

The CD4+ count and plasma viral load values for P1 and P2 are shown in Figure 1. Despite the same exposure, P1 and P2 followed different clinical courses. P1 maintained a CD4+ T cell count greater than 350 cells/mm^3 ^during the first 310 days of untreated infection compared with P2, who had only two CD4+ readings greater than 350 cells/mm^3 ^over the first 249 days of infection. The plasma viral load for P1 was consistently lower than P2 after day 96, with the exception of a second peak reading in P1 taken on day 249, after the detection of HIV-1 super-infection and acute HCV infection. Therefore, P2 appeared to progress more rapidly than P1.

Further clinical laboratory evidence was consistent with the history of a single donor because the time window for one participant to have infected the other was short. Participants P1 and P2 were both positive for p31 antigen on Western Blot on day 63. Therefore, the minimum estimated time since the onset of detectable viraemia (> 50 copies/ml) of approximately 47.4 days [30,31]. Thus, the estimated maximum pre-viraemic phase for either participant was 15 to 16 days. Since, the estimated pre-viraemic phase for HIV-1 lasts between 7 and 25 days [30-33], one participant could have infected the other only between day 7 and day 9 post-exposure to the third party. However, peak viral load in acutely infected subjects is reached 7 or more days after the onset of detectable viraemia [6,12,34] and the infectiousness of a donor MSM is low if his viral load is 400 copies/ml or less [35]. Therefore, while the laboratory evidence did not exclude this alternative scenario, it was unlikely that one participant infected the other.

### Sequences for phylogenetic analysis obtained from multiple viral genes

**Figure 2:**
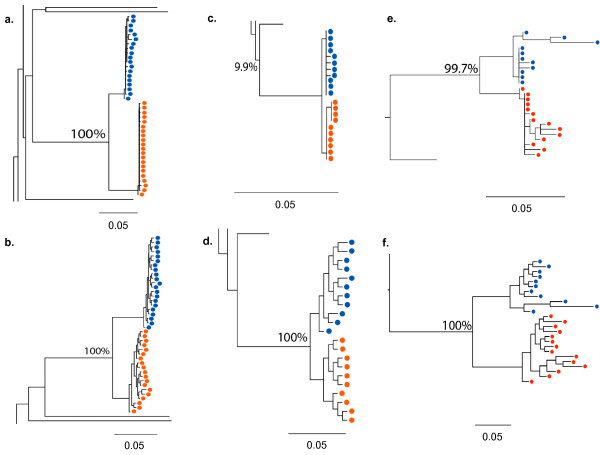
**Trees generated for phylogenetic cluster analysis**. Phylogenetics cluster analysis was carried out using day 63 viral sequences from P1 and P2. Zoomed-in images of trees are shown in Figure 2 for the *env *fragment in a. and b., the *gag *fragment in c. and d., and the *pol *fragment in e. and f.. Results from two different methods of cluster analysis are shown for each fragment: ML (PhyML) trees in a., c., and e., and Bayesian MCMC based consensus trees in b., d., and f.. Terminal nodes represent sequences sampled from P1 (blue circles) or P2 (red circles), as well as reference sequences. *Env *sequences for P1 and P2 were sampled by SGA and represent gap-stripped alignments 1305 nucleotides in length. *Gag *and *pol *fragments were sampled by bacterial cloning. The full tree images can be viewed in Additional Figures 1 and 2. All scale bars show 0.05, equivalent to 5% divergence. ML bootstrap values or Bayesian MCMC based posterior probabilities for the clustering of P1 and P2 are given as percentages next to the common ancestor node.

If P1 and P2 had indeed been infected by the same third person on the same night, we expected that viral sequences sampled from one recipient would be highly similar, or even identical, to sequences sampled from the other recipient. We sampled fragments of three different HIV-1 genes, 63 days post-exposure (Figure 2). The gene fragments were located within the *env, gag *and *pol *genes. We sampled an *env *fragment from the start of the *gp160 *coding region to the end of the *gp120 *coding region (HXB2 nucleotide position 6225 to 7757) by single genome amplification (SGA)[4-6,12,13,36]. After 5% gap-stripping with GapStreeze, the *env *gene fragment alignment was 1305 base pairs in length. The more conserved *gag p24 *to *p6 *(HXB2 1471 to 1976) and *pol Reverse Transcriptase *(*RT*, HXB2 2643 to 3428) gene fragments were sampled by bacterial cloning [37]. We included reference sequences from individuals in the same geographical area and demographic risk group, drawn from the SPARTAC trial and the St Mary's Hospital Acute Infection Cohort [38], as well as the LANL UK reference database. Trees were rooted with outlier sequences from different HIV-1 subtypes and non-M groups in the LANL database. Sequences from both participants clustered with subtype B reference sequences in phylogenetic analyses of all three genes. GenBank accession numbers for sequences from the SPARTAC trial UK cohort and the St Mary's Hospital Acute Infection Cohort in this study are FJ645274 to FJ5645360, JF440652 to JF440693, JF499738 to JF499786, JF506093 to JF506179, and JF692885 to JF693023.

### Between-host phylogenetic analysis supports the clinical history of a single donor

By both maximum likelihood (ML) and Bayesian MCMC based analyses, sequences from P1 and P2 were highly related and clustered to the exclusion of all other sequences, consistent with a common donor (Figure 2, Additional Files 1 and 2). We demonstrated the statistical support for the robustness of the cluster by both methods (Figure 2 - ML bootstrap values for three genes were: *env *100%, *gag *99.9% and *pol *99.3%, and Bayesian MCMC based posterior probabilities were: 100% for *env, gag *and *pol*). We could not use phylogenetic inference to exclude the possibility that one participant infected the other, since such techniques cannot prove the direction of transmission in a forensic sense [39]. For example, we could not exclude the possibility that two strains were transmitted to one participant and that an initially infectious strain was out-competed to extinction prior to day 63. However, results from other studies suggested this was unlikely [5,6,13,40,41]. Therefore, phylogenetic analyses were consistent with the clinical history that a single, third party contemporaneously transmitted the divergent strains that infected P1 and P2.

### Significant between-host divergence observed in transmitted HIV-1 *env *and *pol *genes

We measured the inter-host distance for stem branches, which are the internal branches separating the within-patient sequences. For the *gag *gene fragment, which we expected to be the most conserved fragment, the inter-host distance was 0.54% by ML analysis (Figure 2c). The inter-host distance for the *env *fragment, which we expected to be the least conserved of the three, was 3.81%(Figure 2a). For the *pol *fragment, the inter-host distance was 1.93% (Figure 2e). The inter-host distance for *env *contrasts with the smaller mean distance within each participant. For *env*, the mean within-patient distance was 0.54% by ML analysis in both participants across the gap-stripped 1305 nucleotide alignment, consistent with the history of recent infection (Figure 2a). In addition, sequence analysis of day 235 plasma also failed to detect *env *or *pol *sequences from P1 in P2 and vice versa (data not shown). Therefore, despite sharing highly similar *gag *genes, consistent with the clinical history of a common origin, P1 and P2 appeared to be infected with remarkably different *env *variants and, to a lesser extent, *pol *variants.

**Figure 3:**
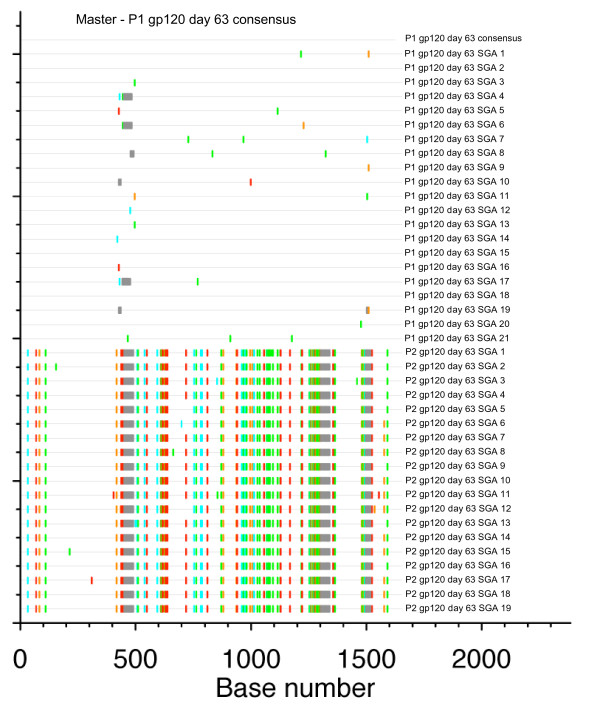
**Highlighter plot of *env gp120 *nucleotide sequences**. Full-length *env gp120 *sequences from day 63 were sampled by SGA. The Highlighter plot shows gaps in grey and nucleotide substitutions (A = green, T = red, G = orange, C = light blue), revealing difficult-to-align regions. The master sequence against which all other sequences are compared is the majority-rule P1 consensus sequence at day 63, shown as the top sequence.

Current implementations of ML and Bayesian tree analysis do not model gaps or non-aligned regions informatively [42]. As phylogenetic analysis of the *env *region meant removing gaps and non-aligned portions, we compared full-fragment, non-stripped *env *sequences from P1 and P2 with the baseline consensus sequence for P1 in a Highlighter plot (Figure 3). There was sequence homogeneity within both P1 and P2, compatible with a single strain initiating a recent infection for each. However, there were multiple sites of variation when P1 was compared with P2. Secondly, we quantified the percentage phylogenetic signal-to-noise (STN)[43] in *env*. We compared our full *env *fragment with gaps to the same fragment with 5% gap-stripping. The percentage STN between P1 and P2 was 70.7% to 24.3% in the unstripped *env *fragment and 62.0% to 30.7% for the stripped *env*. Nevertheless, the percentage STN in the stripped alignment between hosts was greater than in previous studies of multiple-variant transmissions in this genomic region [6,12]. Our analyses indicated that there was a small loss of between-host phylogenetic signal in *env *by stripping gaps or poorly aligned regions. However, stripped *env *fragment alignments contained a higher percentage STN than either the shorter *gag *alignment (49.4% to 50.5%) or shorter *pol *alignment (4.2% to 35.5%). The gag and pol fragment alignments did not require stripping. Noise ≥ 30% was consistent with a phylogenetic cluster [44,45], but we needed to quantify between-host evolution prior to transmission by another method.

### *Env *divergence quantified by estimating the tMRCA

**Figure 4:**
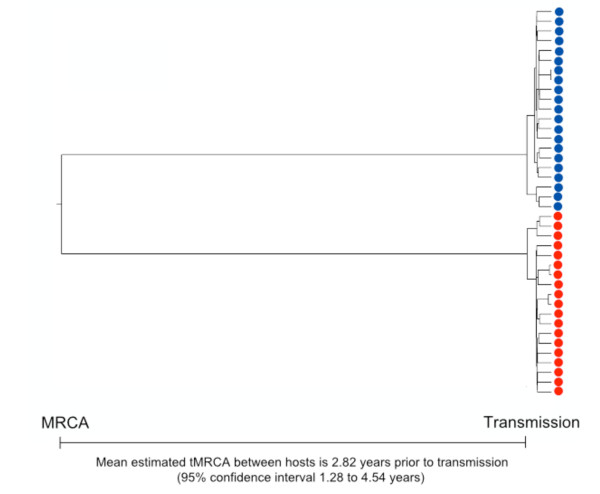
**Relaxed-clock tree for *env***. Between-host divergence, in terms of pre-transmission evolution, was quantified as the estimated tMRCA using a Bayesian MCMC based approach. *Env C2V5 *fragment sequences from P1 and P2, sampled at day 63 by SGA, were calibrated against within-host divergence since the estimated time since transmission as well as the mean rate of substitution from the reference dataset.

To quantify pre-transmission evolution, we estimated the time since divergence of the two *env *variants infecting P1 and P2 by calibrating the sequence evolution rate for the *env C2V5 *region of *gp120 *against another dataset and by measuring the degree of within-host diversification since transmission [3,15]. Using Bayesian MCMC based inference, we estimated the inter-host distance as the time to the most recent common ancestor (tMRCA) which was 2.82 years (95% confidence interval: 1.28 to 4.54 years) of viral evolution (Figure 4). We repeated this analysis with different priors (Additional File 3). All of these results were consistent, and the common ancestor of the HIV-1 *env *genes infecting P1 and P2 was estimated to have existed at least 1.14 years prior to transmission, either in a chronically infected donor or in a recent previous host. These estimates were again consistent with the clinical history of a single, third party having infected both P1 and P2, and that highly divergent sequences could be transmitted by a single donor within a very short period of time.

### Potential antigenic variation in the gp120 proteins of transmitted viruses

However, demonstrating a high level of divergence did not answer whether each patient received divergent variants at random or whether there was selection at transmission. Transmission of divergent *env gp120 *variants could be due to hard selection for differences in antigenicity in each recipient. Hard selection involves selective mortality of variants [46]. In rhesus macaques, SIV envelope proteins appear be under hard selection at transmission due to neutralizing antibodies [47]. Attempts have been made to infer the antigenicity of HIV-1 envelope proteins to neutralizing antibodies from the number of potential N-linked glycosylation sites (PNLGSs) in gp120 [22,48]. Therefore, we hypothesized that differences in the number of PNLGSs in gp120 would indicate potential between-host differences in viral antigenicity.

**Figure 5:**
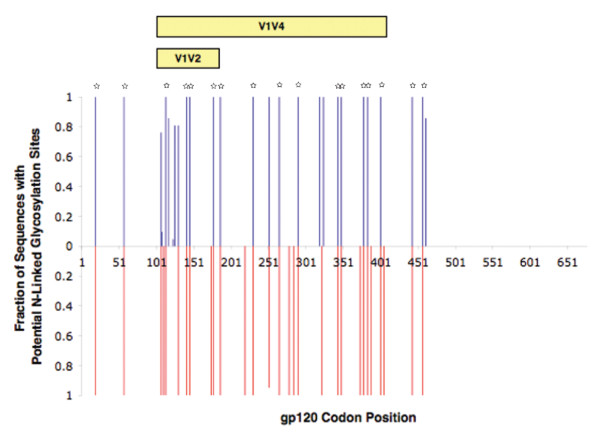
**Comparison of PNLGSs in inferred *env *gp120 amino acid sequences**. Full-length gp120 amino acid sequences, inferred from day 63 SGA nucleotide sequences, are shown. The proportion of P1 sequences with PNLGS at a particular position are shown as a 'positive' blue bar and the proportion of P2 sequences with a PNLGS is shown as a 'negative' red bar. Positions where 100% of sequences have and PNLGS in both P1 and P2 are indicated by small stars.

We compared PNLGSs within inferred amino acid sequences for gp120 from P1 and P2 using N-Glycosite (Figure 5). P1 had a mean of 24 PNLGSs (range 23 to 25). P2 had a mean of 29 PNLGSs (range 28 to 29). Firstly, we looked for positions where P1 and P2 were identical. P1 and P2 shared PNLGSs in 100% of sequences at 17 positions. To demonstrate that this degree of identity was consistent with a phylogenetic cluster, we compared these sequences with 242 unrelated sequences. We studied 87 full-length, inferred amino acid sequences for gp120 sampled from other SPARTAC participants at trial baseline by population sequencing, as well a 155 subtype B sequences from the LANL database sampled during acute infection. The combined SPARTAC/LANL reference sequences had 100% PNLGS predictions at only one site, located in C1. Greater than 90% of the reference sequences had a PNLGSs at only seven positions. We concluded that the degree of similarity between P1 and P2 was consistent with a phylogenetic cluster due to transmission from a single donor.

We then looked at the positions that were not 100% identical, to see if there was any evidence of potential hard selection in each recipient during transmission. In particular, we focussed on the V1V4 region that is implicated in susceptibility to neutralizing antibodies. Previous studies of this region have suggested that fewer PNLGSs in this region increases the susceptibility of highly related strains to neutralizing antibody [22,24,25,49]. We found a higher mean number of PNLGSs across V1V4 in P2 (24 sites, range 23-25) than P1 (19 sites, range 18-20; p < 0.0001, unpaired T-test). These data indicated that there could be a difference in susceptibility to neutralizing antibodies between these two strains, consistent with a non-random model of transmission.

### No autologous or cross-neutralization observed despite potential antigenic variation

We hypothesized that differences at PNLGSs might equate to differences in neutralization that would explain the transmission of divergent *env *variants [22,24,25,49]. Therefore, we investigated whether the viral isolates from P1 and P2 had different neutralization profiles. Viruses pseudotyped with full-length day 63 *env *sequences from P1 and P2 were tested against autologous or heterologous serum from each participant sampled at day 186 post-exposure. However, the *env *pseudotypes for both P1 and P2 were only poorly neutralized or cross-neutralized (half maximal inhibitory concentration, IC_50_, of serum ≤ 1:20, Additional File 4). Therefore, it seemed unlikely that a humoral response was responsible for the detection of different *env *variants in P1 and P2, consistent with transmission being a random process.

However, envelope proteins are not only potentially under immune selection at transmission but also might be selected for an increased ability to enter cells. We used the data from our neutralization assay to estimate the infectivity of the env pseudotyped viruses *in vitro*. Pseudoviruses derived from P1 sequences were approximately 2.5 times (P < 0.05) more infectious *in vitro *than pseudoviruses from P2, after normalization to reverse transcriptase levels (Additional File 5). We noted between-host diversity in *C2C4*, including differences in glycosylation. *C2C4 *encodes discontinuous regions involved in CD4 and co-receptor binding [50-52]. Inferred gp120 protein sequences were analysed with several algorithms that were evaluated by Low and colleagues [53], to detect differences in predicted co-receptor usage and minimize the possibility of missing CXCR4/CCR5 dual-use variants. However, these algorithms predicted that all sampled viruses from P1 and P2 would use CCR5. Our experiment was not specifically set up to test infectivity so all these results must be interpreted with caution. In addition, potential differences in infectivity do not explain why both viruses were able to cause productive infection in different individuals. Therefore, we found no evidence to reject a random model of transmission.

### HLA Class 1 restricted responses and potential selection pressure around transmission

We also investigated HIV-1 specific cellular immune responses, to exclude another potential source of hard selection in each participant that might influence our results. Clinical progression and viral load have been associated with host HLA Class I type in chronic infection [54-56]. HLA Class I restricts the ability of host cytotoxic T lymphocytes (CTLs) to recognize and destroy infected cells. Furthermore, sequencing studies have detected evidence consistent with escape from CTL responses within weeks of HIV-1 infection [57]. The role that CTLs play in preventing established viral infection in humans remains unclear. However, vaccination of rhesus macaques to produce detectable CTL responses is associated with partial protection from infection [58], and HIV-1 specific CTL responses have been detected in persons who remain PCR/ELISA negative despite high-risk exposure [59-61]. Therefore, we hypothesized that CTL responses during and after transmission were a potential source of hard selection in P1 and P2.

Firstly, we compared the Class I HLA type of P1 and P2 with the clinical data to see if there was evidence of selection. P1 possessed HLA-A*0201, A*2402, HLA-B*1402, B*3543, Cw*0102, Cw*0802; P2 possessed HLA-A*0101, A*2901, B*0801, B*5001, Cw*0602, Cw*0701. Neither participant possessed HLA types that are strongly associated with protection from progression in chronic infection [62,63]. However, P2, who progressed quickest, possessed the HLA-A*0101 B*0801 haplotype that is associated with more rapid progression [64]. Therefore, we hypothesize that host factors contribute to the different clinical outcome in these participants and that the viruses had been under different selection pressures since transmission.

### Detectable CTL responses do not explain between-host divergence in *env*

We investigated whether different CTL responses could have influenced detection of divergent variants in our study. Phylogenetic analysis assumes neutral evolution rather than natural selection [44]. Therefore, we compared viral sequence data and γ-interferon ELISpot data from each participant to see if cytotoxic T lymphocyte responses since transmission may have accounted for observed between-host divergence in *env *[65,66]. Sequence data were available for the two env gp120 optimal peptides against which P2 had a significant response: TVYYGVPVWK (HXB2 gp160 30-46) and SFEPIPIHY (HXB2 gp160 202-221). The inferred amino acid sequences for P1 were identical to the wild-type peptides at these epitopes: TVYYGVPVWR and SFEPIPIHY. P2 was also infected with wild-type TVYYGVPVWR, as well as both wild-type and mutant SFEPIPIHK sequences. Therefore, between-host genetic differences in *env *could not be attributed to detectable, env-directed CTL responses, and our data were still consistent with transmission of *env *variants being a random process.

## Conclusions

We have quantified for the first time significant, between-host genetic divergence in HIV-1 variants that are likely to have been transmitted by a single donor to two recipients on the same night. Furthermore, these data indicate that currently it is not possible to predict which of the many HIV-1 variants circulating at the time of transmission will successfully seed a new infection. If transmission is a random process, then this represents a major hurdle that any HIV-1 vaccine design will need to overcome.

## Methods

### Participants

360 participants, 151 of whom were from the UK or Ireland, were recruited to the Short Pulse AntiRetroviral Therapy at HIV seroConversion (SPARTAC) trial (ISRCTN number 76742797; EudraCT number 2004-000446-20). Two male individuals from the UK cohort, P1 and P2, were identified on clinical history as having epidemiologically-linked infections: they were partners and had shared a sexual encounter with a single, third male on the same night. P1 and P2 were enrolled in the trial on the same day and followed up at the Jefferiss Trust Clinic, St. Mary's Hospital, Paddington, London, UK. They were both randomized to receive no therapy.

### Ethics Statement

This study has been approved by the Multicentre Research Ethics Committee (MREC). All participants provided written informed consent before participating in this study.

### HLA typing

Participant HLA type was determined to the oligo-allelic level using Dynal RELITM Reverse Sequence-Specific Oligonucleotide kits for the HLA-A, -B and -C loci (Dynal Biotech). To obtain four-digit typing, Dynal Biotech Sequence-Specific priming kits were used, in conjunction with the Sequence-Specific Oligonucleotide type.

### Separation of PBMCs and plasma

Peripheral blood mononucleocyte (PBMC) and plasma samples were separated from fresh EDTA blood by Ficoll/Hypaque density gradient centrifugation. For PBMC collection, blood was diluted with R10 solution: RPMI 1640 (Sigma UK) with 10% fetal calf serum (FCS; Sigma, UK), 50 units/ml penicillin/streptomycin mix and 2 μM L-glutamine. The mixture was then layered over Lymphoprep separation medium (Gibco, UK). Samples were centrifuged at 100 × *g *at room temperature. The resultant layer of PBMC was removed and washed. 1 ml aliquots containing 5 × 10^6 ^cells were stored in cryotubes in liquid nitrogen at -180±C. For plasma collection, blood samples were prepared as above with dilution with R10, and the resulting plasma was collected in 1 ml aliquots and stored at -80±C.

### Viral RNA extraction

1 ml aliquots of frozen plasma were used for each extraction. The plasma was centrifuged at 1600 × *g *and 4±C for 1 hour to pellet the virus. Excess plasma was removed and the pellet was resuspended in 140 μl of remaining plasma. RNA was then extracted with the QIAamp Viral RNA Minikit (Qiagen, UK) according to the manufacturer's instructions.

### Reverse transcription and polymerase chain reaction (PCR)

For *env*, viral RNA was reverse transcribed using the SuperScript III Kit (Invitrogen, UK) to produce cDNA. 15 μl of viral RNA was added to 1.5 μl dH_2_O, 1.5 μl primer OFM19 [6] (concentration 20 μM) and 1.5 μl dNTPs (concentration 10 mM). The mix was heated to 65°C for 5 min followed by 4°C for 1 mins to anneal the primers to the RNA. The reverse transcription (RT) reaction mix (5xBuffer: 6 μl, DTT: 1.5 μl; RNaseOUT 1.5 μl; SuperScript III 1.5 μl) was then added to make a final volume of 29 μl. The reaction mix was heated to 50°C for 60 min, followed by 55°C for 60 min and finally 75°C for 10 minutes. For *gag *and *pol*, viral RNA was reverse transcribed using the Reverse-iT 1^st ^Strand Synthesis Kit (Abgene, UK). 18 μl of viral RNA was added to 1.5 μl primer (random decamers and oligodT supplied with the kit, concentration 20 μM). The mix was heated to 75°C for 5 min followed by 4°C for 2 min to anneal the primers to the RNA. The RT reaction mix (5×Buffer: 6 μl; dNTPs: 3 μl concentration 10 mM; RTase Blend 1.5 μl) was then added to make a final volume of 30 μl. The reaction mixture was heated to 42°C for 60 min followed by 75°C for 10 min. The HIV *gag *and *pol *genes were amplified by separate PCR reactions as described in detail elsewhere [67]. The HIV *env *genes were amplified by PCR using a protocol for single genome amplification as described in detail elsewhere [5,6].

### Single genome amplification

Single genome amplification (SGA) of *env *was carried as described elsewhere [5,6]. A 30% cut-off for positive wells was used [5,6,36].

### Bacterial cloning

Bacterial cloning was carried out for *gag *and *pol *using the TOPO TA "One Shot" Cloning Kit for Sequencing (Invitrogen, UK). Purified PCR products were ligated into the pCR4-TOPO vector. *Escherichia coli *were mixed on ice with the ligation mix and then transfected by heat shock at 42°C for 30 s. Cells were immediately removed to ice and then added to SOC medium (Invitrogen, UK) and placed on a shaking incubator at 37°C and < 1 × *g *for 1 hour. Cells were then spread on plates of 1× lysogeny broth (LB) agar (Sigma, UK) containing 0.1 μ g/ml ampicillin (Sigma, UK) and incubated overnight at 37°C. Negative controls were included. Colonies were then selected and added to individual wells containing 2× LB medium (Sigma, UK) with 0.05 μ g/ml kanamycin (Sigma, UK). The wells were incubated on a shaking incubator overnight at 37°C and < 1 × *g*. Bacteria were lysed and minipreps of clonal plasmid DNA (pDNA) were prepared using the Montage Miniprep_96 _Kit (Millipore, US).

### Sequencing

Sequencing of population PCR, SGA and bacterial cloning DNA products was performed using BigDye technology in a 96-well plate. For population PCR and SGA products, 3 μl DNA was added to a mix containing 0.8 μl BigDye Terminator (Applied Biosystems, UK), 1.5 μl 5× sequencing buffer (Applied Biosystems, UK), 2 μl of primer (3.3 μM) and 2.7 μl dH_2_O. For bacteria-cloned pDNA, 4 μl of miniprep was added to a mix containing 1 μl BigDye Terminator, 1.5 μl 5× sequencing buffer, 1 μl of primer (3.3 μM) and 3.5 μl dH_2_O. The following cycling conditions were used: 96°C for 30 s, then 30 cycles of 96°C for 30 s, 50°C for 15 s and 60°C for 4 min. DNA for sequencing was precipitated on ice with 2 μl 3M sodium acetate, 10 μl dH_2_O, 50 μl ice-cold 100% ethanol for 5 min at -20°C, centrifuged at 600 × *g *for 80 min at 4°C, washed twice with ice-cold 70% ethanol and run on an ABI 3700 sequencer.

### Sequence alignment

All sequences were manually edited using Sequencher v4.8 (Gene Codes Corporation, US) and manually aligned using Se-Al v2.0a11 [68,69]. For *env *alignment, sequences were first aligned with MUSCLE v3.7 [70] followed by manual alignment. Sequences containing stop codons or frameshifts were deleted prior to subsequent analysis. Where appropriate, reference sequences were obtained from the Los Alamos National Laboratory (LANL) HIV sequence database [71]. For *env*, which contains many gaps and poorly aligned regions, gap stripping was undertaken first with GapStreeze set to 5% [72]. In GapStreeze, the user sets a gap tolerance between 0% and 100%. A value of 5% will cause all columns in the alignment to be deleted if more than 5% of sequences contain a gap at that position. Sequences were manually edited in Se-Al v2.0a11 before and after gap-stripping.

### Between-host phylogenetic analysis

Phylogenetic analysis of viral sequences sampled from P1 and P2 was carried out by several methods across the *env, gag *and *pol *gene-fragments. Prior to gap-stripping with GapStreeze, a likelihood mapping [45] analysis was run to ensure phylogenetic signal within *env *was significant. Likelihood mapping was implemented in Tree-Puzzle v5.3.rc7 [73] and the *env *fragment was screened from the beginning of the coding start region to the end of *gp120 *(HXB2 nucleotide position 6225 to 7757). Additionally, full nucleotide sequences for the fragments from all three genes were visually screened in Highlighter [74], and the inferred protein sequence were screened visually using Jalview v2.6 [75,76]. Phylogenetic trees were initially constructed using the maximum likelihood (ML) method with PhyML v3.0 software [77], and visualized in FigTree v1.3.1 [78]. We chose the substitution model that gave the highest likelihood with PAUP*v4.0 [79]: the generalized time reversible (GTR) model incorporating estimates of the proportion of invariant sites (I), and the shape parameter of a gamma distribution [80]. ML branch support values were obtained by non-parametric bootstrapping using PhyML v3.0 (1000 replicates). Finally, phylogenetic analysis using a Bayesian MCMC based method was implemented in Mr Bayes v3.1.2 [81,82]. An unconstrained branch length (exponential) prior was used to avoid enforcing a molecular clock [44]. MrBayes v3.1.2 was run in duplicate for at least 50,000,000 steps for *env *and *pol*, sampling trees every 1,000 steps. MrBayes v3.1.2 was run in duplicate for at least 100,000,000 steps for *gag*, sampling every 10,000 steps. Convergence was assessed with Tracer v1.5 [83] with all parameter estimates having effective sample sizes (ESSs) of > 300, because a high ESS reflects a low degree of correlation among samples [44]. The consensus tree for each gene, with posterior probabilities for branch support, was generated and visualized in FigTree v1.3.1.

### Inferring the tMRCA using a relaxed molecular clock

To determine the time to the most recent common ancestor (tMRCA) of the sequences isolated from the two participants, we used a Bayesian MCMC based approach. We tested our assumption that all of the observed evolution in *env *within the viral sequence sets from each participant had occurred within each host by demonstrating a star-like intra-host phylogeny, and confirming that intra-host divergence by ML was consistent with that predicted for early, monophyletic infection against other datasets [3,5,6,12,15]. We used a normal tMRCA prior for the sequences within each participant, calibrated to a mean of 63 days since exposure (standard deviation 1 day). We ran BEAST v1.5.4 [84] for at least 100,000,000 steps, sampling every 10,000 steps, and employing an uncorrelated lognormal relaxed clock to allow for rate variation among branches [15,44,85-87]. Rate variation may occur if the two variants evolved at different rates, before or after transmission [15,44]. The substitution model was the GTR model. The underlying demographic model was the Bayesian skyline plot with 10 steps, and was used as a flexible prior on the distribution of the inter-node intervals on the sampled phylogenetic topologies [15,44,85-87]. Convergence was assessed with Tracer v1.5, and all parameter estimates had ESSs of > 300 [15,44,85-87]. Convergence was not achieved when using estimated transmission time as the only prior; the ESSs for the prior and posterior probabilities remained < 100 after 300,000,000 steps [15,44,85-87]. To deal with this issue, a posterior mean rate of substitution prior was estimated from the posterior mean rate of another dataset, for a fragment of the *env C2V5 *region [15]. This mean rate prior was normally distributed, with a mean of 8.18 × 10^-3 ^substitutions per site per year (standard deviation of 1.15×10^-3 ^substitutions per site per year) [15]. The hypervariable regions were cut to be consistent with the original dataset after consultation with the authors [88]. To achieve convergence, our relaxed-clock analysis also required the full-length *C2V5 *fragment, rather than the part-fragment used in the reference dataset that was missing the 5' end of the *C2 *region [15].

To determine the sensitivity of our results to the choice of prior, we also analysed the data under a strict molecular clock, calibrating the time of transmission to the same prior as under the relaxed molecular clock, but not enforcing a strong prior on the rate [15,44]. We performed this analysis for *C2V5 *and our entire 1305 stripped *env *fragment. The mean rate prior from the reference dataset was necessary for the, relaxed clock analysis to converge, but our tMRCA estimate was robust to this choice of prior, as the most important prior for the tMRCA estimate was the time of transmission. Although calibration to the time since transmission may lead to an overestimate of the posterior substitution rate estimate [15], other studies have found that this effect is small for monophyletic infections [6,12]. Both strict-clock and relaxed-clock analyses using the *gag *and *pol *fragments failed to achieve convergence after 300,000,000 steps, and no reference datasets were available for calibration of evolution in these fragments.

### Potential N-linked glycosylation site analysis

We compared potential N-linked glycosylation sites (PNLGSs) between inferred amino acid sequences for the SGA samples *env *in from P1 and P2, using N-Glycosite [89].

### Neutralization and infectivity assays

HIV *env *genes were amplified from reverse transcribed viral RNA, restriction-cloned in pcDNA3.1 (Invitrogen, UK) and co-transfected into 293T cells with an *env *deficient backbone, nl4.3Δenv (Dr M. Pizzato, University of Geneva). Virus-containing supernatants were harvested, assayed for reverse transcriptase activity [90], and titrated onto the HIV permissible cell-line, TZM-BL (also known as JC53-BL) using previously described techniques [91] with the following modifications: cell monolayers were fixed with 0.2% gluteraldehyde, stained with an X-gal substrate and air dried. Infected cells were counted with an AID v2.9 EliSpot plate-counter (AID GmbH, Germany). To test serum-mediated neutralizing responses, 400 focus forming units (FFUs) of titrated-pseudovirus were incubated with serial dilutions of heat inactivated autologous sera from participants. Neutralization was calculated as the percentage-reduction of FFUs compared to virus-only controls.

### IFN-γ ELISpot assay

100 μl of 0.5 μ g/ml mouse anti-human IFN-γ monoclonal antibody solution (Mabtech, Sweden) was added to each well on an ELISpot plate (Millipore, US). Frozen PBMCs were rapidly defrosted and then pipetted into 10 ml of a solution containing RPMI 1640 and pig skin gelatine (PSG) with added DNAse (Sigma, UK). The solution was centrifuged at 300 × *g *for 5 min. The PBMCs were resuspended in 20 ml of R10 solution and incubated overnight at 37±C. Cells were then counted and resuspended in a volume of R10 solution to give a final concentration of 5 × 10^5 ^cells per 100 μl. The ELISpot plate was washed three times with 200 μl per well of phosphate buffered solution (PBS; Gibco, US) containing 1% FCS. Peptides were added to the appropriated wells, with a final concentration of each peptide being 10 μM. We used overlapping 15 mer peptides covering HIV-1 proteins gag p17 and gag p24 as well as optimal epitopes covering gag, pol, nef and env proteins. 100 μl of PBMC suspension was then added to each well. Duplicate negative controls were prepared, containing R10. Duplicate positive controls were prepared, containing 5 μ g/ml PHA-P (Sigma, UK). The plate was incubated for 16 hours at 37±C. The PBMCs were then discarded and the plate was then washed seven times with PBS. 100 μl of 0.5 μ g/ml biotinylated anti-human IFN-γ monoclonal antibody (Mabtech, Sweden) was added to each well. The plate was incubated for 90 min at room temperature. The antibody was then discarded and the plate washed seven times with PBS. 100 μl of 0.5 μ g/ml streptavidin-conjugated alkaline phosphatase (ALP; Mabtech, Sweden) was added. The plate was incubated at room temperature for 40 min. The streptavidin-ALP was then discarded and the plate washed seven times with PBS. 100 μl of substrate solution from the ALP conjugate substrate kit (Bio-Rad, US) was added to each well. The plate was incubated at room temperature for 10 min, or until a colour change was noted in the positive control well. The plate was then washed with ordinary tap water and dried. Spots were counted on the AID version 2.9 EliSpot plate-reader. The normalized magnitude of the response (*NMOR*) was calculated as follows [92]:

Where *M*_exp _is the number of spots in the experimental well,  is the mean number of spots in the negative control wells, and *SD_neg _*is the standard deviation of the negative control wells. *NMOR *is always a positive integer and all negative values are set to 0.

## Competing interests

The authors declare that they have no competing interests.

## Authors' contributions

RP and JF conceived the study; JF, SE and AK designed the study; SE, DB, PF and AD performed the experiments; SE, AK and DB analysed the data; SE and JF wrote the paper; RP, MM, JW, SF and STSC contributed participant information and samples; all authors were involved in drafting this paper; all authors have read and approved the final manuscript.

## Supplementary Material

Additional file 1 **Images of the entire ML (PhyML) trees for a. *env*, b. *gag *and c. *pol***. Terminal nodes representing day 63 sequences sampled from P1 (blue circles) and P2 (red circles), as well as reference sequences are shown. *Env *sequences for P1 and P2 were sampled by SGA and represent gap-stripped alignments of full-length *gp120*. *Gag *and *pol *fragment sequences were sampled by bacterial cloning. Click here for file

Additional file 2 **Images of the entire Bayesian MCMC based consensus trees for a. *env*, b. *gag *and c. *pol***. Terminal nodes representing day 63 sequences sampled from P1 (blue circles) and P2 (red circles), as well as reference sequences are shown. *Env *sequences for P1 and P2 were sampled by SGA and represent gap-stripped alignments of full-length *gp120*. *Gag *and *pol *fragment sequences were sampled by bacterial cloning. Click here for file

Additional file 3 **Robustness analysis for Bayesian MCMC based approach for a. *env C2V5 *and b. the entire *env *fragment**. The tMRCA estimation analysis was repeated for *env *SGA sequences using a strict molecular clock. The estimated time since transmission was the only prior. Click here for file

Additional file 4 **Neutralization assay results**. Neutralization assay results are shown for day 186 post-exposure sera from P1 and P2 against pseudoviruses typed with day 63 P1 and P2 envelopes. Results for two clones from each participant are shown for both autologous and cross-neutralization assays at two serum dilutions, 1:20 and 1:60. Click here for file

Additional file 5 **Results of the infectivity assays**. Infectivity assays were used to titre pseudoviruses prior to infection for the neutralization assay. Fold virus dilutions are shown in the legend. The results for the two clones used in the assay shown in Additional File 4 are shown but these results were consistent for the nine clones screened for each participant. Infectivity is corrected against viral reverse transcriptase expression. Click here for file
