# Supplementary figures and images for: Phylogenetic analysis consistent with a clinical history of sexual transmission of HIV-1 from a single donor reveals transmission of highly distinct variants
Source: Retrovirology. 2011 Jul 7;8:54. doi: 10.1186/1742-4690-8-54 (PMC3161944; doi:10.1186/1742-4690-8-54)

a.

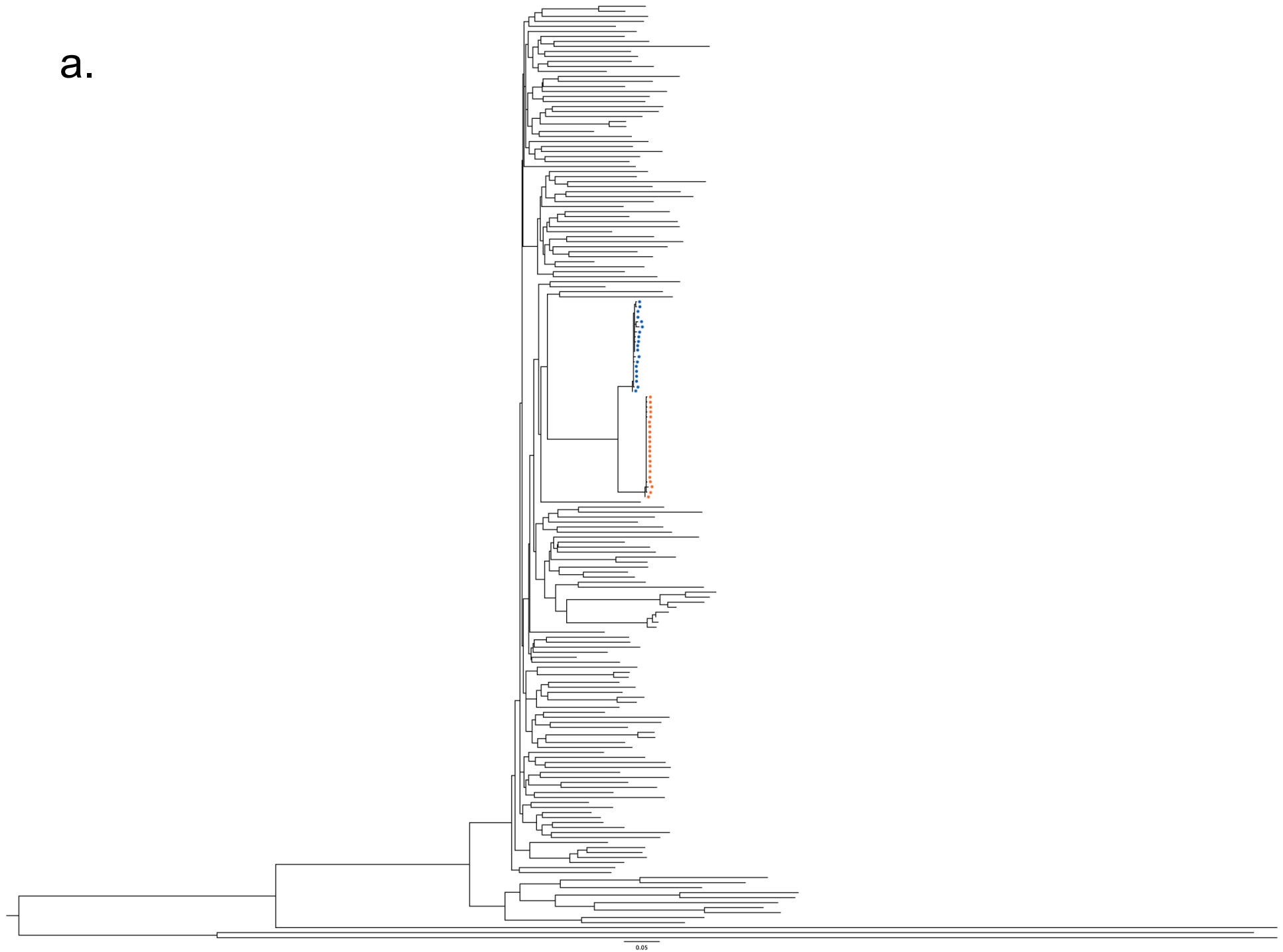

b.

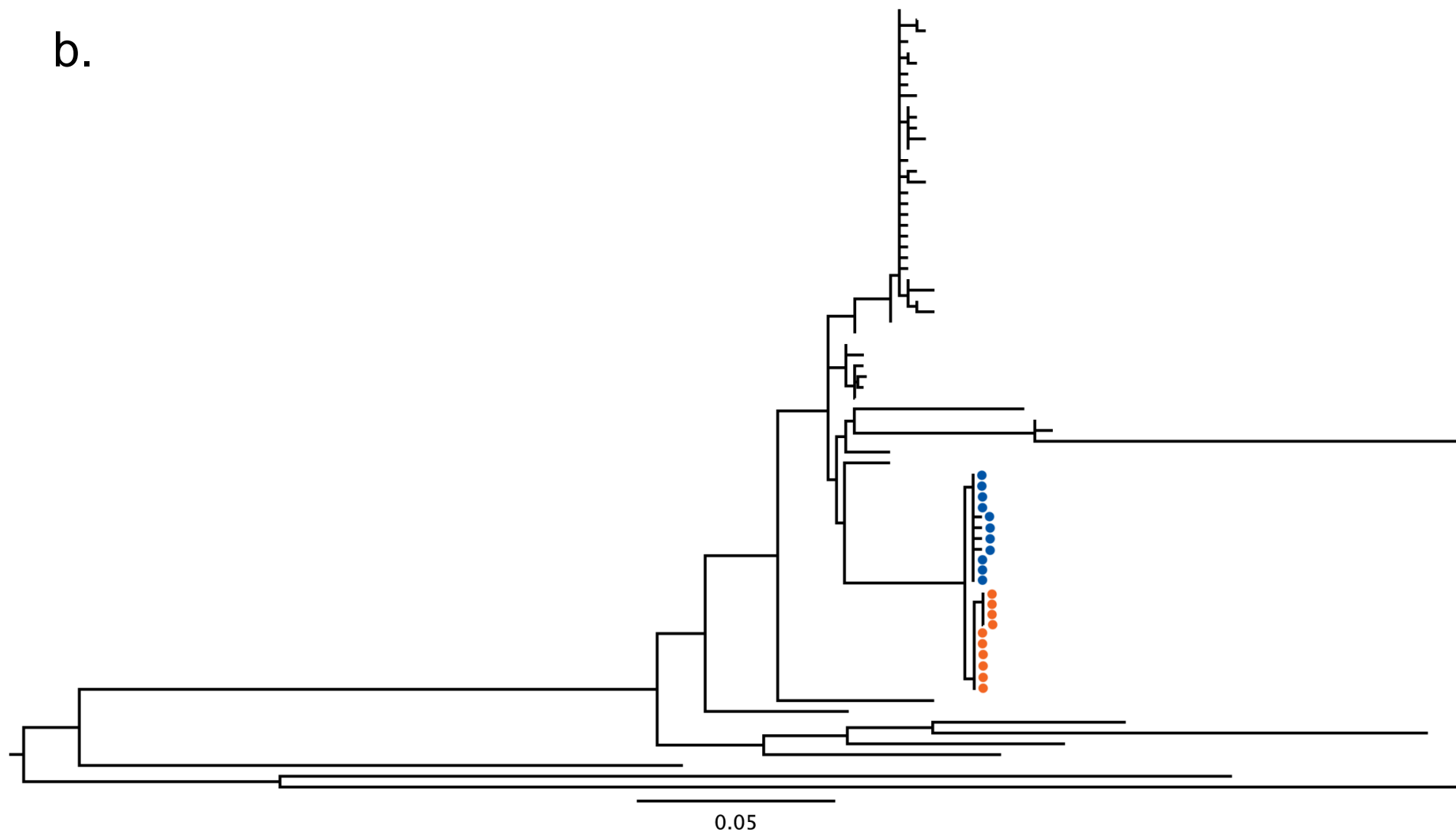

C.

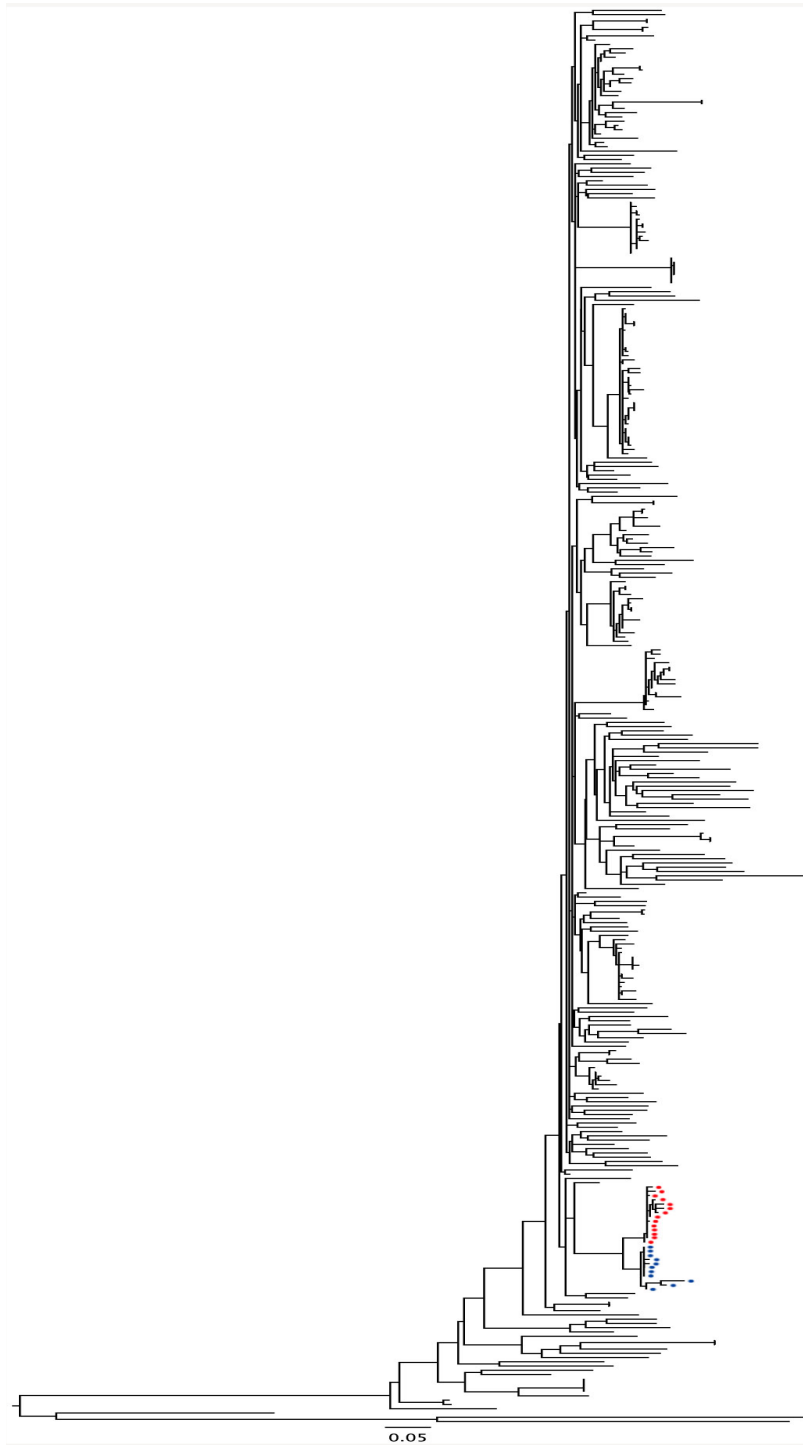

Supplement: Additional file 1 — Images of the entire ML (PhyML) trees for a. env, b. gag and c. pol. Terminal nodes representing day 63 sequences sampled from P1 (blue circles) and P2 (red circles), as well as reference sequences are shown. Env sequences for P1 and P2 were sampled by SGA and represent gap-stripped alignments of full-length gp120. Gag and pol fragment sequences were sampled by bacterial cloning. [file 1742-4690-8-54-S1.PDF]

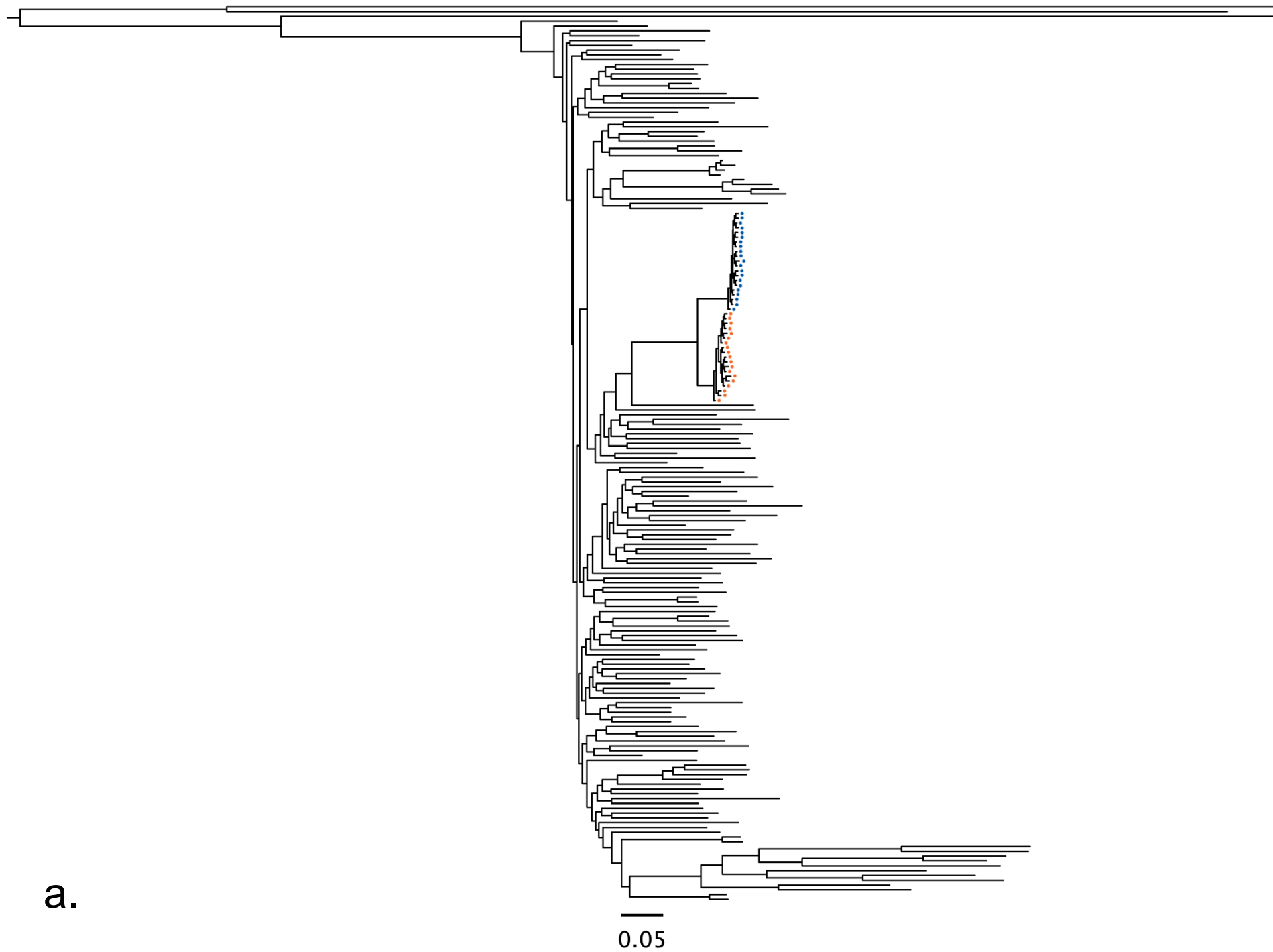

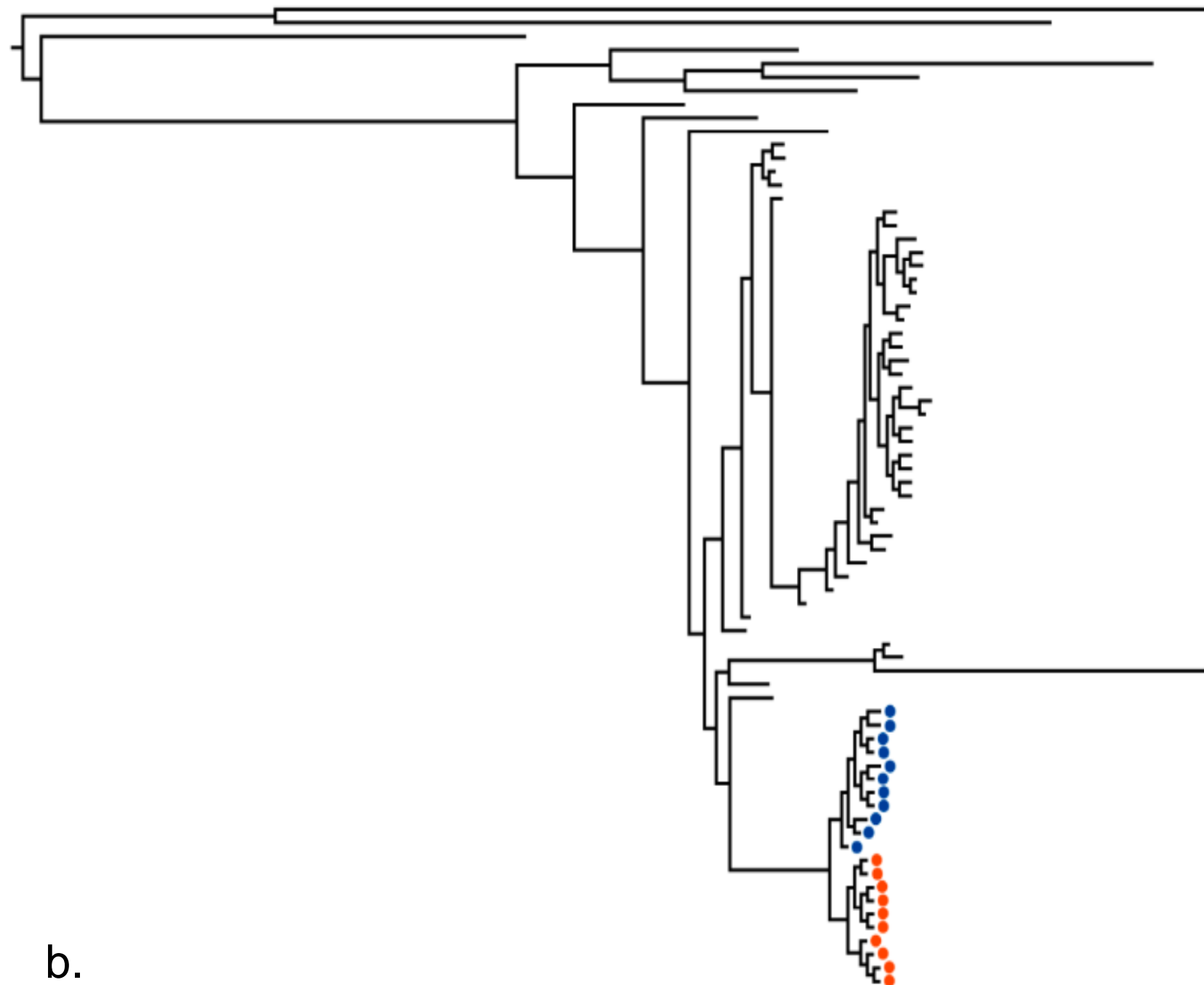

0.05

b.

C.

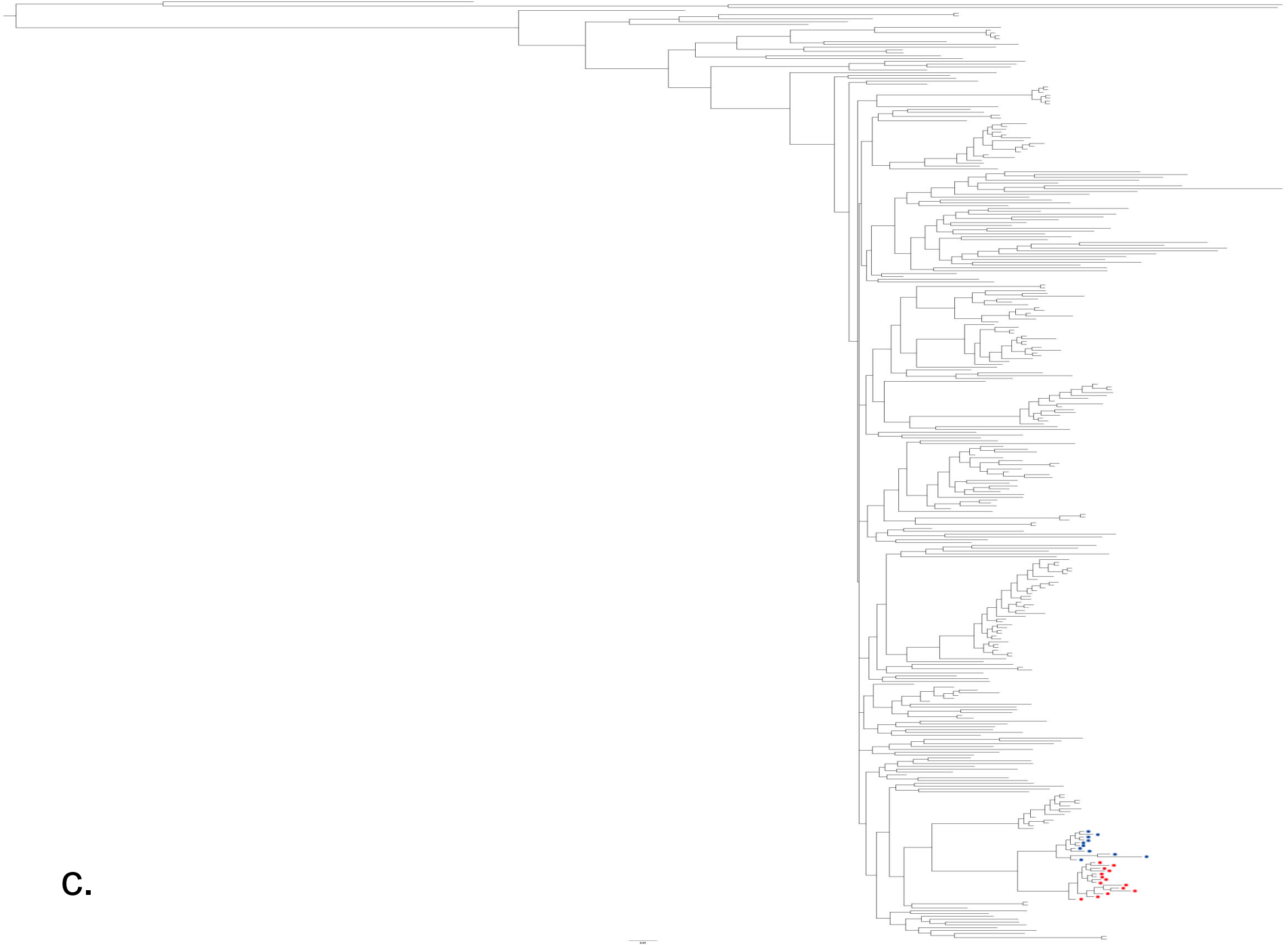

Supplement: Additional file 2 — Images of the entire Bayesian MCMC based consensus trees for a. env, b. gag and c. pol. Terminal nodes representing day 63 sequences sampled from P1 (blue circles) and P2 (red circles), as well as reference sequences are shown. Env sequences for P1 and P2 were sampled by SGA and represent gap-stripped alignments of full-length gp120. Gag and pol fragment sequences were sampled by bacterial cloning. [file 1742-4690-8-54-S2.PDF]

**Pseudovirus P1 Clone 1**

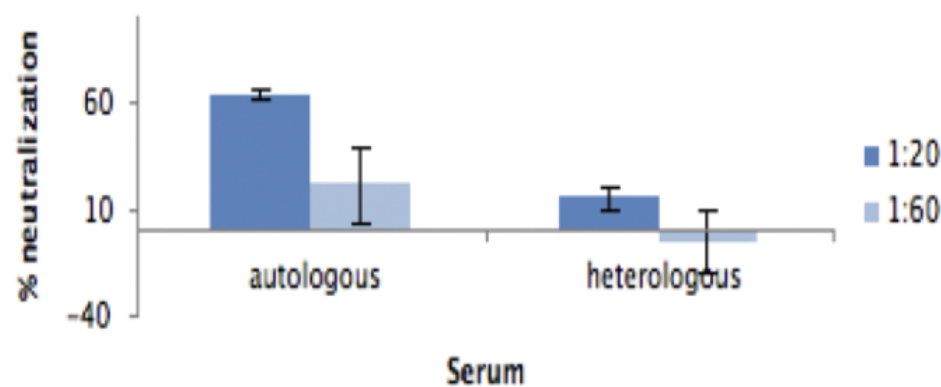

**Pseudovirus P1 Clone 2**

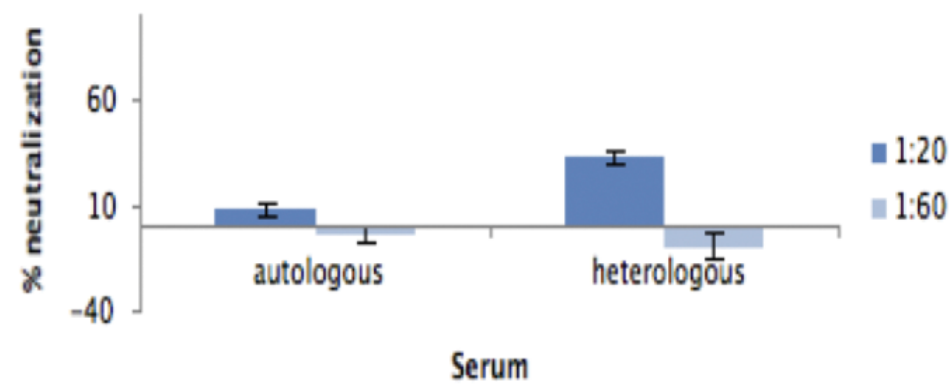

**Pseudovirus P2 Clone 1**

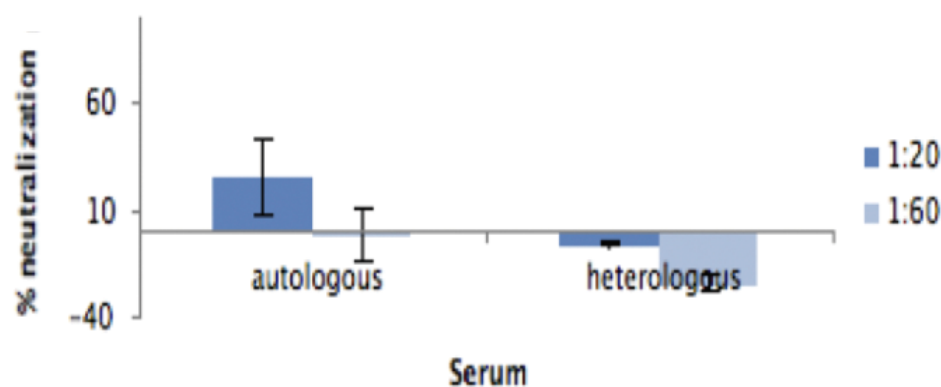

**Pseudovirus P2 Clone 2**

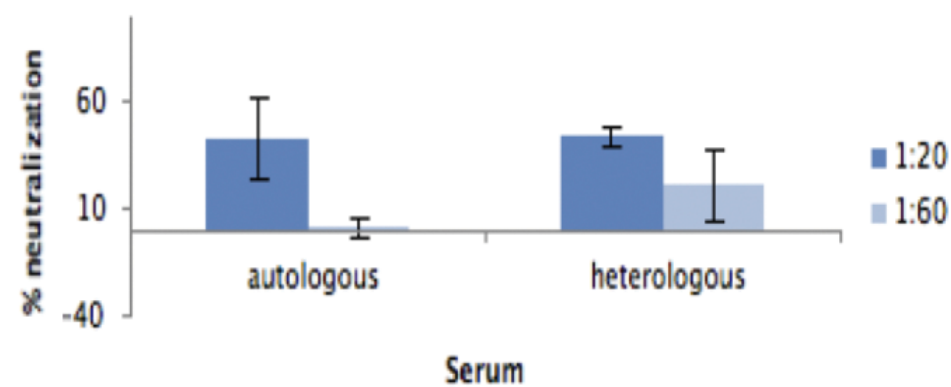

Supplement: Additional file 4 — Neutralization assay results. Neutralization assay results are shown for day 186 post-exposure sera from P1 and P2 against pseudoviruses typed with day 63 P1 and P2 envelopes. Results for two clones from each participant are shown for both autologous and cross-neutralization assays at two serum dilutions, 1:20 and 1:60. [file 1742-4690-8-54-S4.PDF]

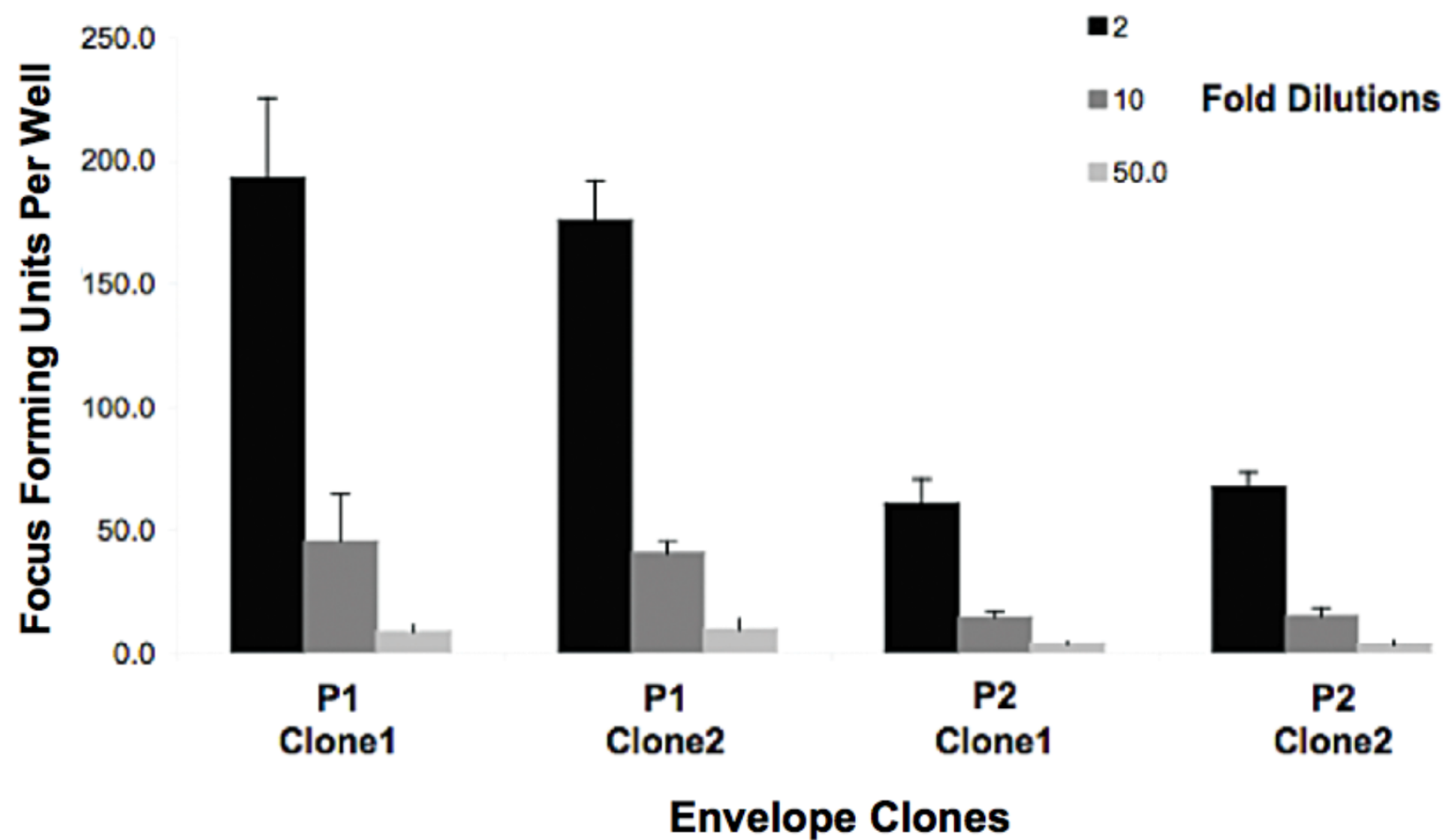

Supplement: Additional file 5 — Results of the infectivity assays. Infectivity assays were used to titre pseudoviruses prior to infection for the neutralization assay. Fold virus dilutions are shown in the legend. The results for the two clones used in the assay shown in Additional File 4 are shown but these results were consistent for the nine clones screened for each participant. Infectivity is corrected against viral reverse transcriptase expression. [file 1742-4690-8-54-S5.PDF]
