# Supplementary material for: Phylogenetic analysis consistent with a clinical history of sexual transmission of HIV-1 from a single donor reveals transmission of highly distinct variants
Source: Retrovirology. 2011 Jul 7;8:54. doi: 10.1186/1742-4690-8-54 (PMC3161944; doi:10.1186/1742-4690-8-54)
Supplement: Additional file 3 — Robustness analysis for Bayesian MCMC based approach for a. env C2V5 and b. the entire env fragment. The tMRCA estimation analysis was repeated for env SGA sequences using a strict molecular clock. The estimated time since transmission was the only prior. [file 1742-4690-8-54-S3.PDF]

a. Strict-Clock *env C2V5*

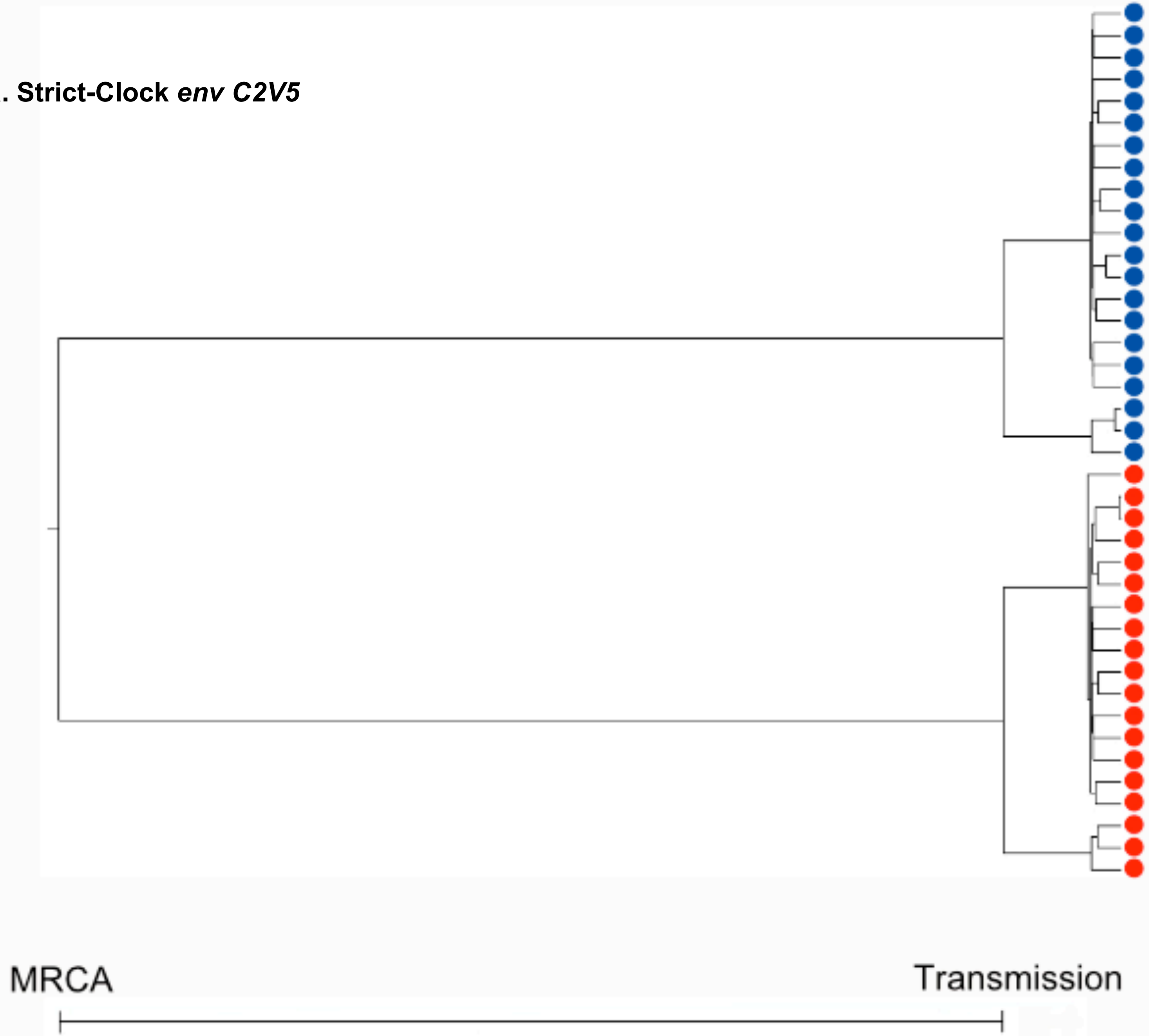

Mean estimated tMRCA between hosts is 2.63 years prior to transmission
(95% confidence interval 1.14 to 4.58 years)

b. Strict-Clock (*gp160* coding start to *gp120* end)

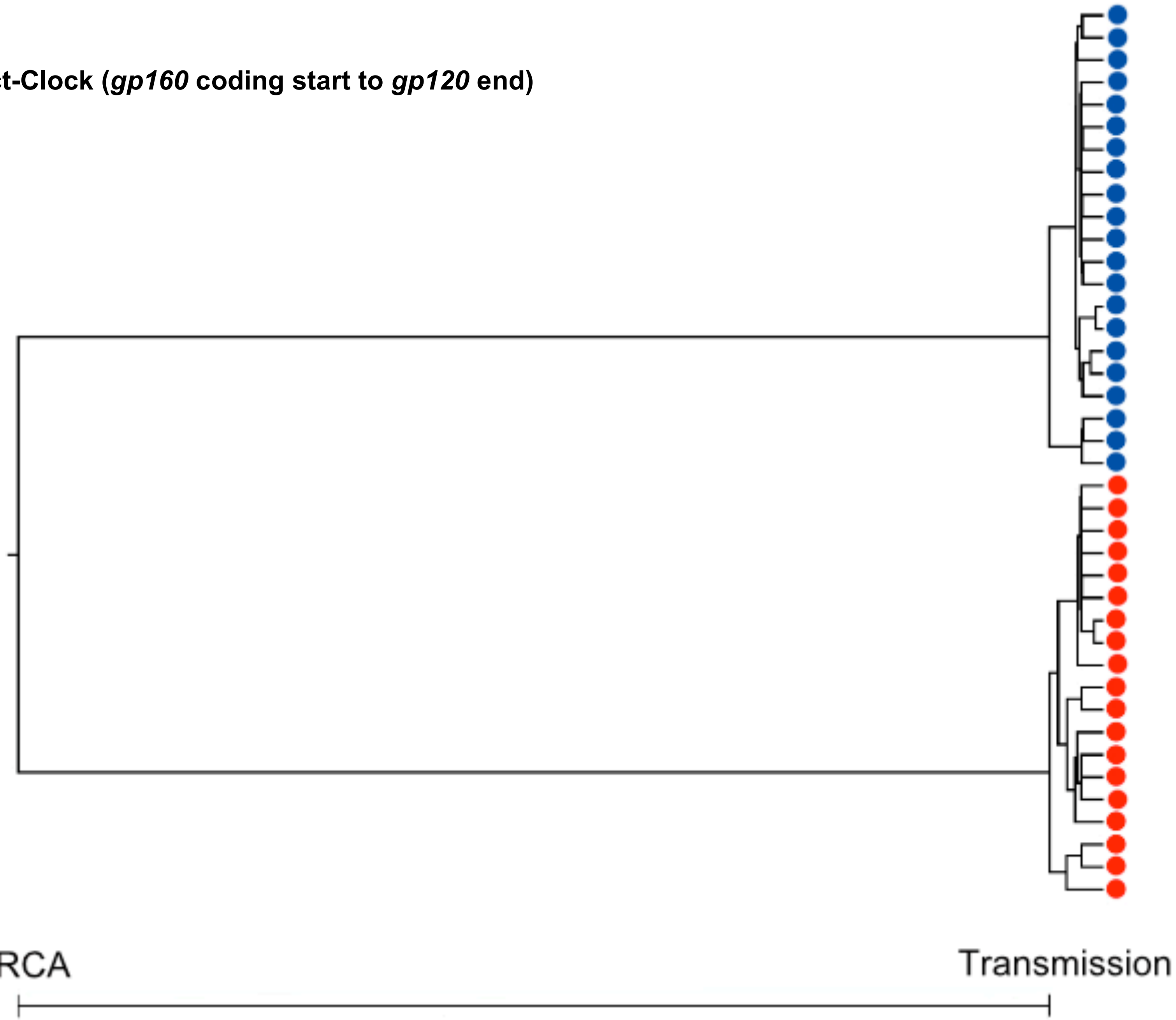

Mean estimated tMRCA between hosts is 2.36 years prior to transmission
(95% confidence interval 1.30 to 3.60 years)
